# Supplementary material for: Cationic Azobenzenes as Light-Responsive Crosslinkers for Alginate-Based Supramolecular Hydrogels
Source: Polymers (Basel). 2024 Apr 28;16(9):1233. doi: 10.3390/polym16091233 (PMC11085219; doi:10.3390/polym16091233)
Supplement: Supplementary file 1 [file polymers-16-01233-s001.zip › polymers-2929432-supplementary.pdf]

Cationic Azobenzenes as Light-Responsive Crosslinkers for Alginate-Based Supramolecular Hydrogels

Miriam Di Martino [1], Lucia Sessa [1,2,*], Barbara Panunzi [3], Rosita Diana [3], Stefano Piotto [1,2] and Simona Concilio [1,2,*]

[1] Department of Pharmacy, University of Salerno, Via Giovanni Paolo II, 132, 84084 Fisciano, Italy; midimartino@unisa.it (M.D.M.); piotto@unisa.it (S.P.)
[2] BIONAM Research Center for Biomaterials, University of Salerno, 84084 Fisciano, Italy
[3] Department of Agriculture, University of Napoli Federico II, Via Università 100, 80055 Portici, Italy; barbara.panunzi@unina.it (B.P.); rosita.diana@unina.it (R.D.)
* Correspondence: lucsessa@unisa.it (L.S.); sconcilio@unisa.it (S.C.)

Supplementary Materials

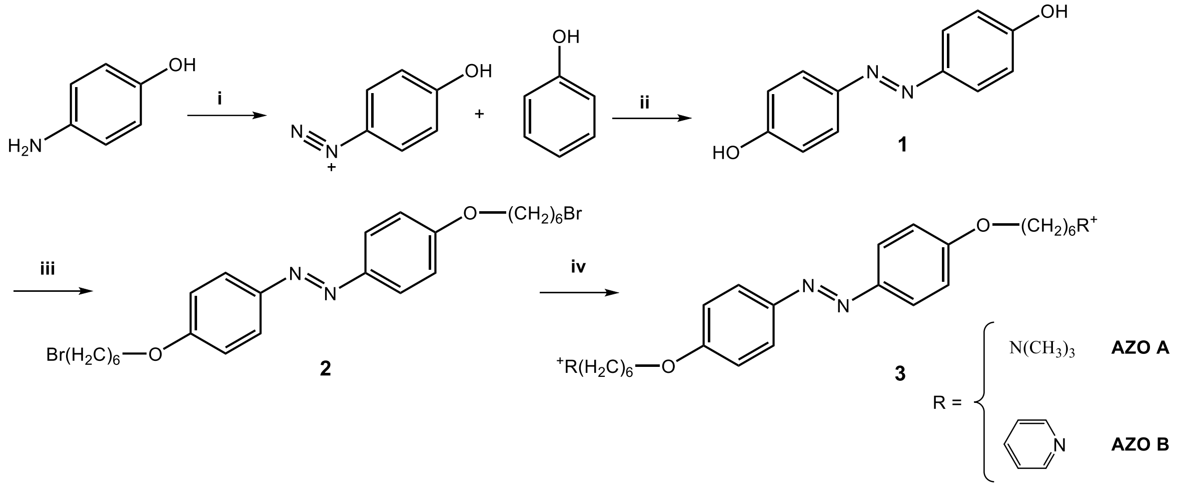

Figure S1. Synthetic scheme of AZO A and AZO B.

i) $NaNO_2$, H_2O/HCl, 0-5°C, 30 min; ii) NaOH pH=11, 10-15°C, 3h; iii) 1,6- dibromohexane, K_2CO_3, CH_3CN dry, reflux, 48h; iv) $N(CH_3)_3$ in ethanol solution, 50°C, 48h for AZO A and Py in acetonitrile, 70°C, 48h for AZO B.

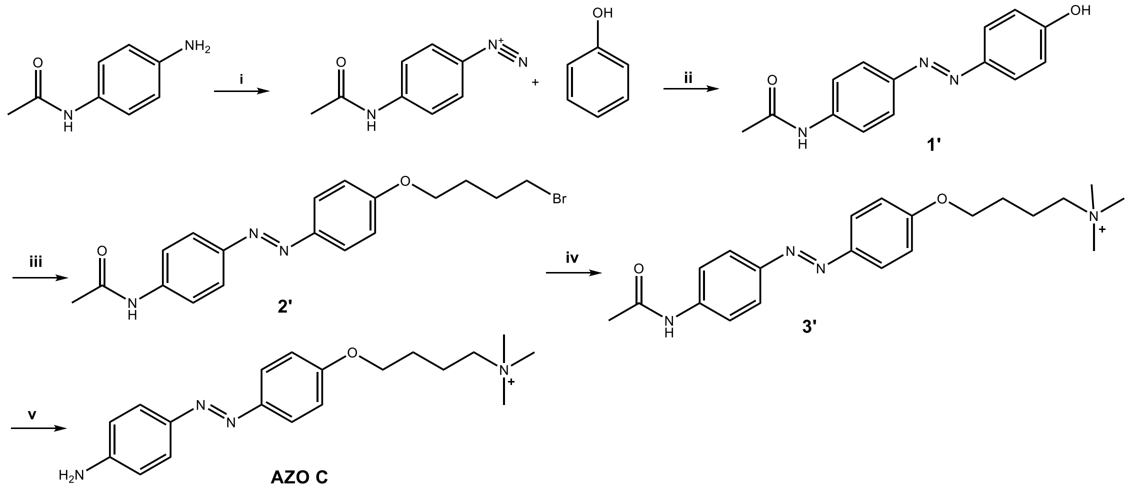

Figure S2. Synthetic scheme of AZO C.

i) $NaNO_2$, H_2O/HCl, 0-5°C, 30 min; ii) NaOH pH=11, 10-15°C, 3h; iii) 1,4-dibromobutane, K_2CO_3, KI, acetone, reflux, 4h; iv) $N(CH_3)_3$ in ethanol solution, 50°C, 48h; v) HCl conc, 40°C, overnight.

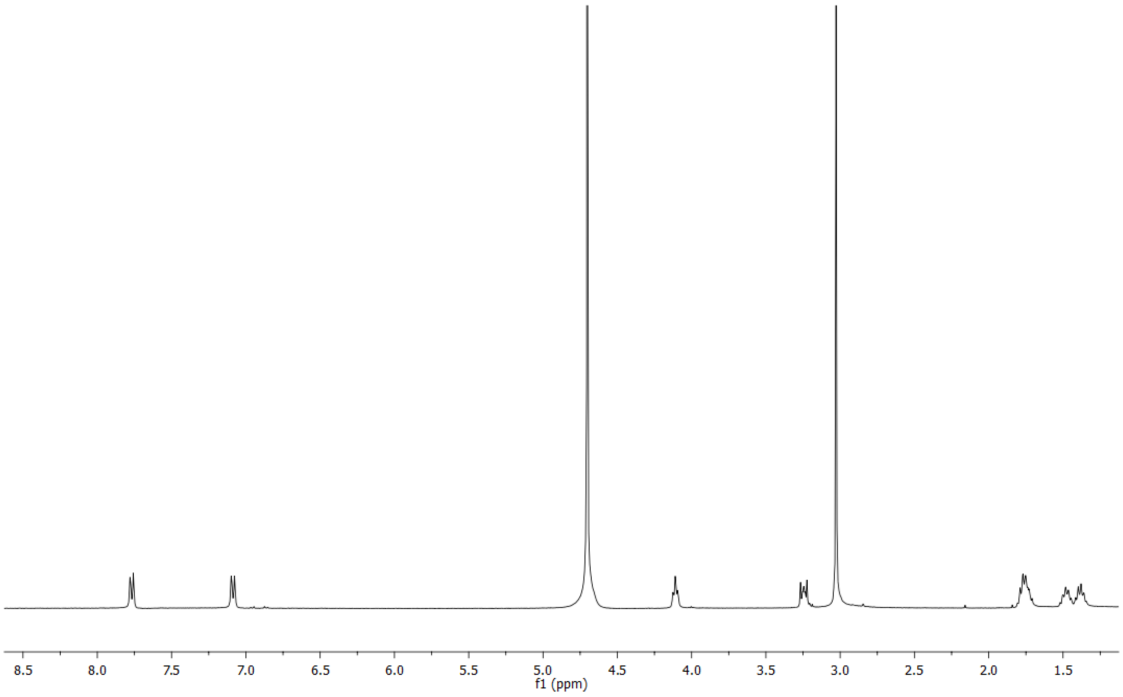

Figure S3. ^{1}H NMR of AZO A (D_2O; 400 MHz)

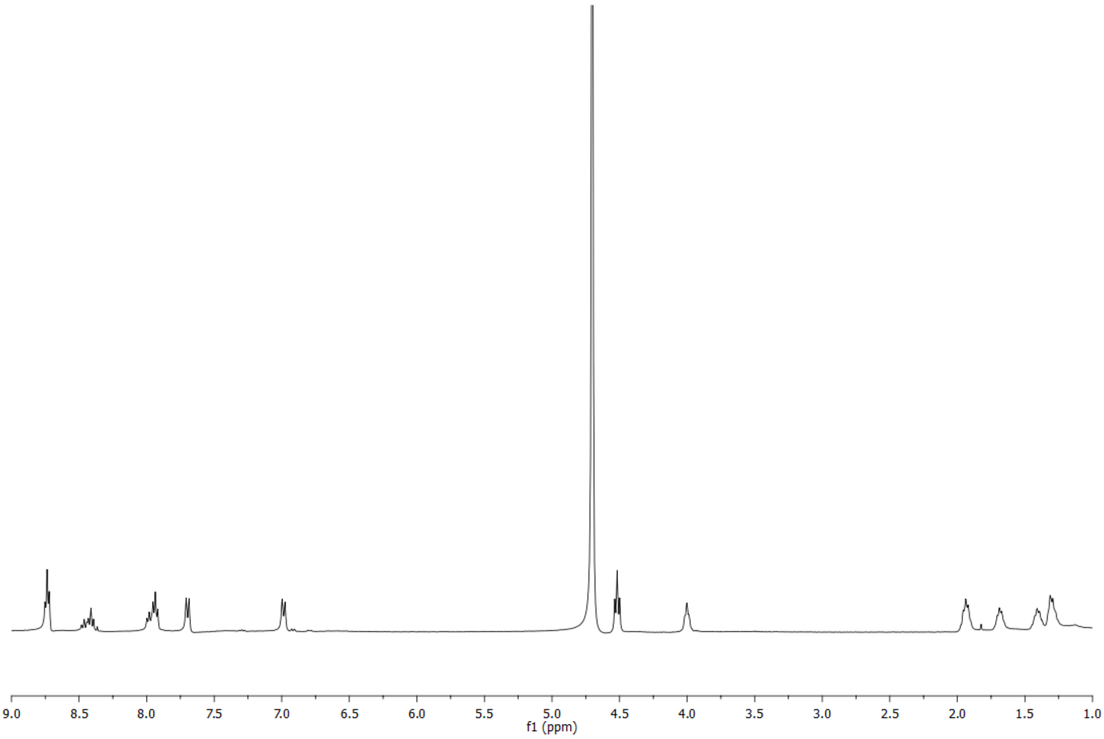

Figure S4. ^{1}H NMR of AZO B (D_2O; 400 MHz)

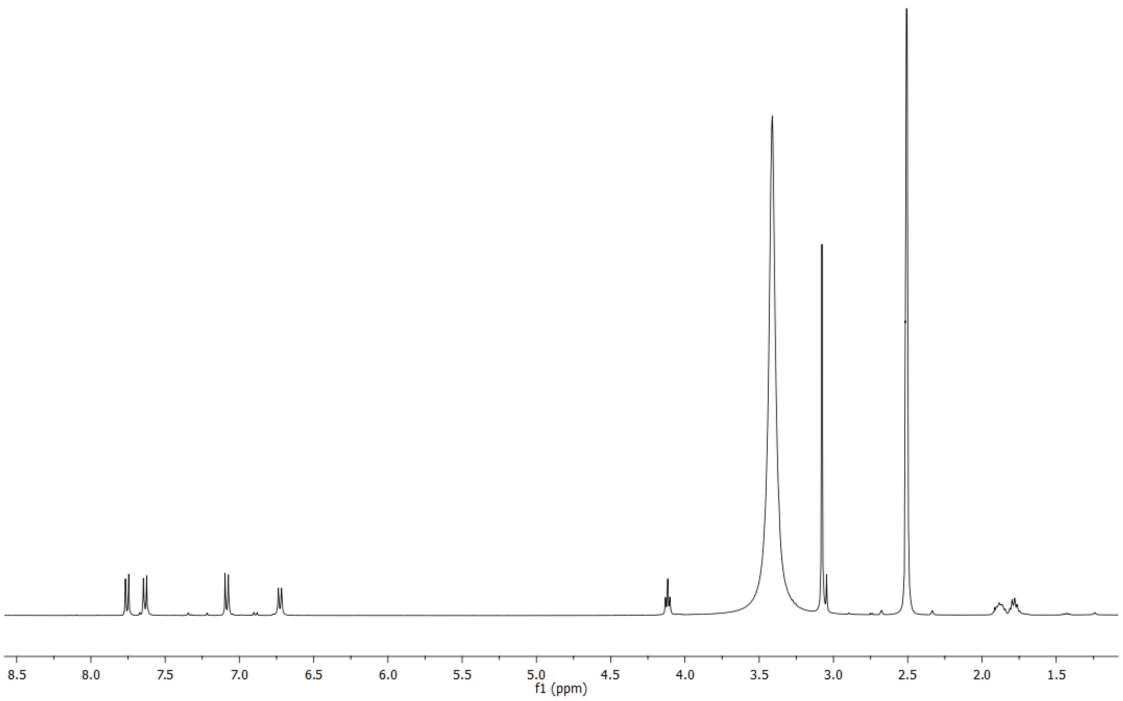

Figure S5. ^{1}H NMR of AZO C (DMSO-d_6; 400 MHz)

Table S1. Scheme of molecular behavior of the hydrogels under the action of pHs and lights

	Composition (wt%)	Hydrogel pH 7	Hydrogel pH 4	Gel-sol (365nm irradiation)	Sol-gel (day light)
**SA_AZO A**	8/0.5	×	✓	✓	✓
	8/1	×	✓	✓	✓
	5/0.5	×	×		
	5/1	×	✓	✓	✓
	2/0.5	×			
	2/1	×			
**SA_AZO B**	8/0.5	×			
	8/1	×			
	5/0.5	×	✓	✓	✓
	5/1	×	✓	✓	✓
	2/0.5	×	×		
	2/1	×	×		
**SA_AZO C**	8/0.5	×			
	8/1	×			
	5/0.5	×	✓	✓	✓
	5/1	×	✓	✓	✓
	2/0.5	×	×		
	2/1	×	×		

Table S2. IR absorption peaks of main functional groups of azobenzenes and corresponding hydrogels

Functional group	AZO A (cm^{-1})	AZO B (cm^{-1})	AZO C (cm^{-1})	SA_AZO A Hydrogel (cm^{-1})	SA_AZO B Hydrogel (cm^{-1})	SA_AZO C Hydrogel (cm^{-1})
**-CH sp^2**	3450-3380	3450-3380	3450-3380	3450-3380	3450-3380	3450-3380
**-OH**				3200 (of SA)	3200 (of SA)	
**$-NH_2$**			3500<br>1650<br>830			
**$-NH_3^+$**						3300-3190<br>1620
**-CH sp^3**	2940-2865	2940-2865	2940-2865	2940-2865	2940-2865	2940-2865
**$-COO^-$**				1600<br>1400 (of SA)	1600<br>1400 (of SA)	1600<br>1400 (of SA)
**-C=C- aromatic**	1598-1578	1600-1400	1504-1480			
**-N=N-**	1492	1492	1547			
**$-CH_3-(N^+)$**	1475					
**-C-N- (C aromatic)**	1238	1242	1379			
**-C-N- (C aliphatic)**	1140	1147	1253	1246<br>(of AZO A)	1250<br>(of AZO B)	
